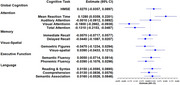# Effects of Diabetes on Cognition and Brain Morphology among Middle‐aged and Older Adults without Dementia from Rural India

**DOI:** 10.1002/alz70860_105371

**Published:** 2025-12-23

**Authors:** SHRUTI PANDEY, Hitesh Pradhan, Ravi S Muddashetty, Jonas S Sundarakumar

**Affiliations:** ^1^ Centre for Brain Research, Indian Institute of Science, Bangalore, India; ^2^ Centre for Brain Research, Indian Institute of Science, Bangalore, Karnataka, India; ^3^ Centre for Brain Research, Indian Institute of Science Campus, Bangalore, India

## Abstract

**Background:**

Diabetes is a known risk factor for dementia. However, the effect of diabetes on cognitive functioning and brain morphology among non‐demented individuals, particularly in the rural Indian population is less explored. This study aims to examine the association between diabetes, cognitive performance and MRI brain structure among community dwelling middle‐aged and older adults from rural India.

**Method:**

This cross‐sectional study utilized baseline clinical, biochemical, and cognitive data of participants aged ≥45 years (*n* = 3,983, mean age=58 years) without dementia from the Centre for Brain Research ‐ Srinivaspura Aging, NeuroSenescence, and COGnition (CBR‐SANSCOG) study cohort, an ongoing prospective cohort study in rural southern India. Diabetes was diagnosed based on clinical history and/or HbA1c ≥6.5%. Cognitive performance was assessed across multiple domains using the culturally adapted COGNITO (Computerized assessment of adult information processing) battery. A subset of these participants (*n* = 1,336) underwent 3T brain MRI (Siemmens Magnetom Prisma scanner), and voxel‐based morphometry (VBM) analysis was performed on the T1‐weighted images. Multivariate linear regression analyses were conducted separately for cognitive and neuroimaging outcomes, adjusting for age, sex, education, body mass index (BMI), tobacco use, alcohol use, hypertension, and depression.

**Result:**

Individuals with diabetes exhibited significantly poorer cognitive performance in the attention domain, particularly in the visual attention and mean reaction time tasks (*p* < 0.05) compared to individuals without diabetes. Additionally, higher HbA1c levels in these individuals were associated with poorer attentional task performance. The VBM analysis revealed significantly reduced grey matter volumes in several regions among individuals with diabetes, including the bilateral cerebellum and right putamen (*p* < 0.05, FWE‐cluster corrected).

**Conclusion:**

This study demonstrates that diabetes is associated with both cognitive impairments in the attention domain alongside grey matter loss in the bilateral cerebellum and right putamen even before the onset of dementia, highlighting the potential adverse impact of diabetes on brain health in rural Indians. Further, worsening glycaemic control among individuals with diabetes was associated with poorer attention. Therefore, early diagnosis and prompt interventions for diabetes could be a scalable and cost‐effective strategy to reduce dementia risk in this population.